# Quantifying Cellular Nanoplastic Accumulation Using Raman Microscopy

**DOI:** 10.1021/acsomega.6c06443

**Published:** 2026-10-22

**Authors:** Federico Perrella, Valentina Iezzi, Roberta Maria Ferri, Giulia Fioravanti, Marilena Briglia, Andrea Marini, Elisabetta Benedetti, Paola Benassi, Carino Ferrante, Davide Tedeschi

**Affiliations:** † Department of Physical and Chemical Sciences, 9303 University of L’Aquila, Via Vetoio, 67100 L’Aquila, Italy; ‡ Life Health and Environmental Sciences, 367958 University of L’Aquila, Via Vetoio, 67100 L’Aquila, Italy; § Department of Medicine and Surgery, 217140 University of Enna “Kore”, 94100 Enna, Italy; ∥ 231873 CNR-SPIN, c/o Department of Physical and Chemical Sciences, Via Vetoio, 67100 L’Aquila, Italy

## Abstract

Micro- and nanoplastic contamination poses emerging health risks through ingestion of contaminated food and water, thereby placing the intestinal barrier as the primary interface for nanoparticle translocation following ingestion. In this context, Caco-2 epithelial cells provide a well-established *in vitro* model of the small intestine barrier, yet label-free quantification of nanoparticle uptake at the single-cell level remains a significant methodological challenge in nanotoxicology. Here, we report a confocal micro-Raman spectroscopy strategy for per-cell quantification of polystyrene nanoparticle (150 nm diameter) accumulation in Caco-2 intestinal epithelial cells. Our approach combines ratiometric analysis, referencing the polystyrene marker band at ∼1002 cm^–1^ to the cellular amide I band at ∼1660 cm^–1^, with control-derived thresholding to distinguish cell-associated nanoparticles from endogenous phenylalanine background, followed by single-cell masking to compute quantitative uptake descriptors. Application to cells exposed to 16 and 25 μg/mL polystyrene for up to 24 h revealed concentration-dependent uptake dynamics. Whereas qualitative spatial occupancy suggests greater nanoplastic accumulation at the lower dose, a rigorous quantification—difficult to achieve with conventional fluorescence-based approaches—uncovers the true dynamics at both concentrations, reconciling apparently paradoxical trends with the complementary transcriptional response and cell viability assays presented herein. These findings demonstrate that the ratiometric Raman mapping framework proposed here provides a chemically specific and robust approach for studying nanoparticle–cell interactions relevant to nanoplastic risk assessment, suggesting that spatial organization of cell-associated nanoparticles as well as relative uptake burden may act as critical determinants of cellular bioactivity.

## Graphical abstract

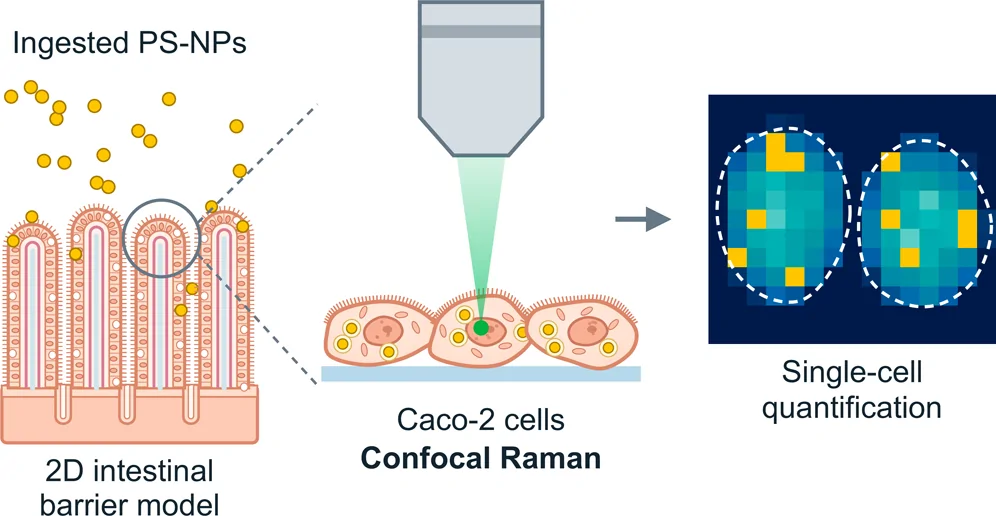

## Introduction

In the last decades, mounting evidence of the ubiquitous presence of micro- and nanoplastics in the environment has raised urgent concerns about their potential impact on wildlife^1–3^ and human health,^4,5^ with evidence of translocation across biological barriers and accumulation in tissues.^6–8^ Micro- and nanoplastics derived from widespread polymers such as polystyrene (PS) are now routinely detected in water, food, and atmospheric samples, and have been reported in biological matrices ranging from lung and liver tissue to breast milk and blood, although the implications of these findings for human physiology remain uncertain.^5,9,10^ Although nonfunctionalized polymers present a macroscopic inert surface in standard bulk culture vessels, their nanoscale, dispersed presentation enables cellular internalization. Owing to their high surface-area-to-volume ratio, internalized nanoplastics can interfere with mitochondrial function and promote reactive oxygen species (ROS) generation, triggering redox-sensitive inflammatory signaling and, upon sustained exposure, delayed cytotoxicity.^11^ However, the exact cellular burden and spatial dynamics governing these toxicological outcomes remain insufficiently quantified. Nanoparticles (NPs) have been widely reported to cross cellular membranes and undergo internalization in various cell types.^12–14^ Thus, understanding how particle size, concentration, and exposure time govern the uptake mechanisms is a central challenge for both nanotoxicology and nanomedicine. In this context, monodisperse synthetic PS spheres are widely used as representative proxies for environmental micro- and nanoplastic contamination. One of the major challenges in nanotoxicology lies in the reliable detection and identification of NPs that have crossed the cell membrane, as well as in tracking their subsequent intracellular trafficking.

Current approaches for the detection and spatial localization of micro- and nanoplastics within cells often rely on fluorescent-labeling imaging.^15–17^ However, while fluorescence microscopy provides high sensitivity and ease of use, the introduction of exogenous fluorescent labels does not reflect native physiological conditions, and fluorescent tags can detach from NPs in the cellular environment, generating spatially diffused signals that do not faithfully represent the true particle distribution.^18,19^ Moreover, the photophysical properties of fluorescent probes introduce additional systematic uncertainties that undermine quantitative reliability. In particular, photobleaching, fluorescence intermittency (blinking), and the sensitivity of fluorescence quantum yield to the local intracellular environment mean that fluorescence signals are not absolute and are prone to artifacts.^20,21^ Fluorescence- and FLIM-based approaches can nonetheless achieve substantially lower limits of detection, down to the sub-μg/mL range: dye-doped nanoparticles combined with phasor-FLIM event counting have been shown to reliably detect PS- and PMMA-based nanoparticles in solution at concentrations as low as ∼0.58 μg/mL.^22^ Nile Red-based fluorescence lifetime imaging further allows discrimination between different plastic polymer types via distinct lifetime fingerprints within the same sample.^23^ This sensitivity advantage, however, relies on the use of exogenous fluorescent labels or dye-doped particles. Label-free spectroscopic techniques such as Fourier transform infrared (FTIR) and Raman spectroscopy provide a direct route to chemical identification of internalized NPs, avoiding the need for exogenous tags. In this context, confocal micro-Raman spectroscopy has emerged as a powerful label-free technique for the identification of polymer micro- and NPs in complex biological matrices like tissues and cultured cells, owing to its intrinsic chemical specificity, high spatial resolution, and higher penetration depth in an aqueous environment with respect to FTIR microscopy.^24–27^ However, most studies up to date have focused on the qualitative detection and localization of PS particles, with quantitative assessment of cellular uptake largely relying on fluorescence-based approaches.^15,28,29^

Intracellular particle quantification using spontaneous Raman is mainly challenged by the intrinsically weak scattering cross section and the spectral overlap between particle markers and cellular components, as well as the lack of a standardized postprocessing workflow able to extract robust metrics from large hyperspectral data sets. To address this gap, we developed a methodological workflow for the localization, identification, and quantification of nonfluorescent PS-NPs in adherent cells with confocal micro-Raman spectroscopic imaging. Here, we report quantitative, single-cell evidence of concentration-dependent uptake kinetics for 150 nm PS-NPs in a Caco-2 *in vitro* intestinal cell model. Using a confocal micro-Raman-based strategy and a tailored data-analysis pipeline, we show that Caco-2 epithelial cells display dose-dependent NP uptake behaviors as a function of their incubation time, providing a systematic, label-free workflow that is directly relevant for risk assessment and nanotoxicology. Specifically, we demonstrate that qualitative assessment of the cellular NP spatial distribution alone is insufficient to resolve concentration-dependent uptake dynamics. Indeed, spatial distribution is informative of cell-associated NP localization but carries no information about their accumulated load, and thus per-cell quantification is essential for the correct interpretation of both spectroscopic and biological results. The biological end points assessed in this study served as a complementary readout to determine whether Raman-detected PS-NP accumulation is associated with early inflammatory modulation, cytotoxicity, or alterations in epithelial integrity.

## Results and Discussion

Caco-2 cells were cultured as adherent monolayers while exposed to PS-NPs under conditions described in Materials and Methods Section, intended to model the intestinal epithelium encountered by NPs following ingestion. Multiple Raman maps were then acquired from an independent well for each combination of PS concentration (16 or 25 μg/mL) and incubation time (6, 15, or 24 h). However, all of the wells are realized simultaneously, changing the persistence in the NP-contaminated environment exclusively. Figure 1 shows representative Raman spectra acquired from isolated PS microparticles (bottom), Caco-2 cells exposed to PS-NPs (middle), and unexposed control Caco-2 cells (top). The pure PS spectrum displays its most prominent feature in the 980–1015 cm^–1^ window (highlighted by the left dashed box), corresponding to the symmetric breathing mode of the phenyl ring, which serves as the primary chemical marker for PS identification throughout this study.^30^ Critically, the control cell spectrum also exhibits a weak but discernible feature at approximately 1002 cm^–1^, arising from endogenous phenylalanine residues in cellular proteins. This spectral overlap represents the central analytical challenge addressed by our ratiometric normalization approach, described below. Nonetheless, this band is substantially enhanced in the PS-exposed cell spectrum relative to controls. In the fingerprint region, cellular spectra display bands at 1250–1320 cm^–1^ (C–N stretching in amide III) and 1450 cm^–1^ (CH_2_ scissoring mode).^31^ The amide I band centered at 1660 cm^–1^ (highlighted by the right dashed box) dominates both cellular spectra, reflecting C=O stretching vibrations in peptide bonds and serving as a ubiquitous protein marker.^31,32^ This band exhibits no intensity in the pure PS spectrum, making it an ideal normalization reference for quantifying PS content relative to cellular biomass.

**Figure 1:**
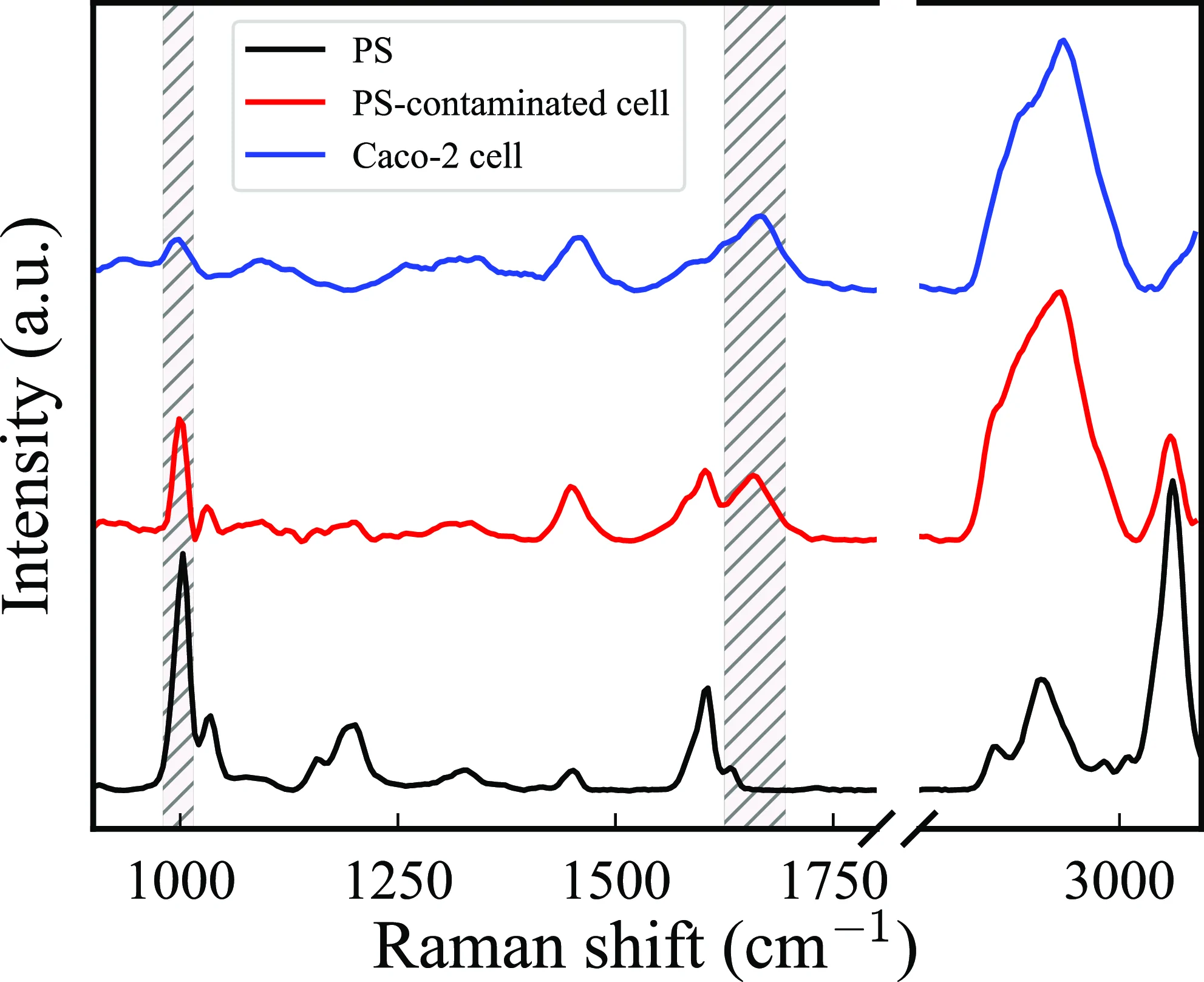
Raman spectroscopic identification of PS-NPs in Caco-2 cells. Representative preprocessed Raman spectra acquired from (bottom) isolated PS microparticles, (middle) PS-NPs contaminated Caco-2 cells, and (top) unexposed control Caco-2 cells. The characteristic PS marker band in the 980–1015 cm^–1^ dashed spectral region (phenyl ring breathing mode) is prominent in the pure PS spectrum and enhanced in PS-exposed cells relative to controls. The control spectrum also exhibits a weak feature at 1002 cm^–1^ attributable to endogenous phenylalanine, demonstrating the spectral overlap challenge addressed by the ratiometric normalization approach. Key cellular bands include the amide I peak in the 1630–1690 cm^–1^ dashed spectral region (protein C = O stretching) and the CH_2_/CH_3_ stretching window at 2850–2950 cm^–1^ (mainly lipids). Spectra are baseline-corrected and vertically offset for clarity.

In the high-frequency region, intense bands appear in all three spectra: peaks at 2856 cm^–1^ correspond to symmetric CH_2_ stretching, the 2888 cm^–1^ band arises from antisymmetric CH_2_ stretching, while bands at 2931 cm^–1^ and 2980 cm^–1^ reflect CH_3_ symmetric and antisymmetric stretching, respectively.^32,33^ Lastly, a sharp feature also appears at 3060 cm^–1^, corresponding to aromatic C–H stretching.

Exploiting the spatial mapping capability of confocal micro-Raman spectroscopy, Figure 2 qualitatively illustrates the subcellular distribution of PS-NPs within Caco-2 cells after 15 h of exposure (25 μg/mL). While conventional optical microscopy (Figure 2A) provides only a general morphological context, chemical mapping of the PS marker band (Figure 2B) highlights the NP distribution as discrete, punctate hotspots. In parallel, the Amide I band map (Figure 2C) accurately defines the cellular footprint based on protein distribution. Finally, the overlaid visualization of the two (Figure 2D), with the PS image rendered with α transparency, confirms that the PS-rich aggregates are strictly localized within the projected cellular boundaries. Such subcellular heterogeneity is consistent with vesicular compartmentalization of internalized NPs and with endocytic uptake mechanisms reported for particles of this size range.^13^ Importantly, this example demonstrates the capability of confocal micro-Raman mapping to simultaneously resolve NP aggregates and the underlying cellular architecture without exogenous labeling, providing the chemical and spatial context required for the subsequent quantitative analysis.

**Figure 2:**
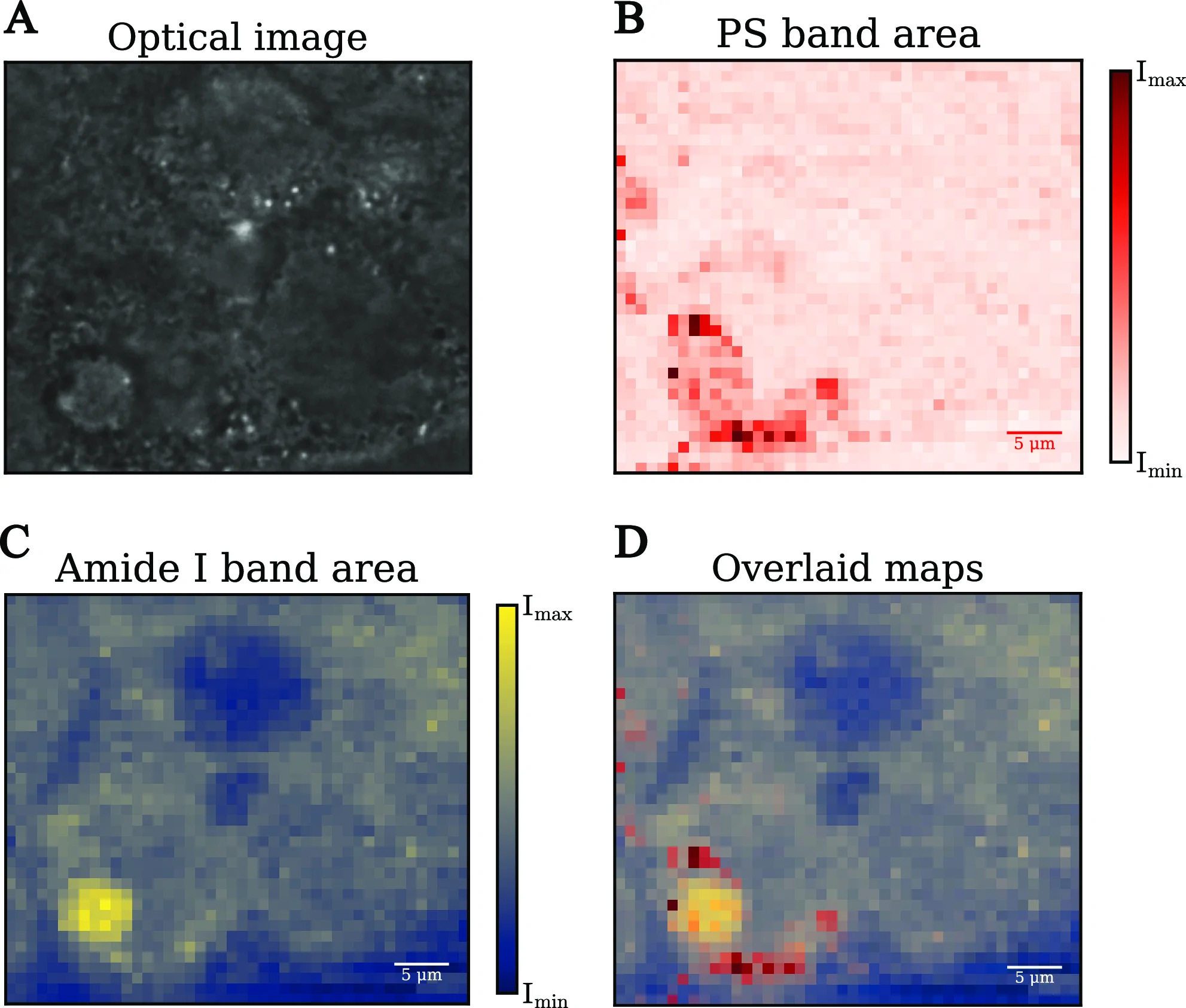
Spatial mapping workflow for subcellular PS-NPs localization. Representative hyperspectral Raman analysis of Caco-2 cells exposed to 25 μg/mL PS-NPs for 15 h. (A) Optical micrograph of the sampled region showing multiple adherent cells with typical epithelial morphology; (B) spatial distribution of integrated Raman intensity in the 980–1015 cm^–1^ window (PS marker band), revealing discrete regions of elevated signal corresponding to NP accumulation; (C) spatial distribution of integrated Raman intensity in the amide I band region (1630–1690 cm^–1^), delineating cellular protein distribution and morphology; and (D) overlaid visualization created by applying α transparency to the PS map and superimposing it on the cellular amide I distribution. Color scales represent integrated band areas in arbitrary units.

It is worth noting that this method does not allow one to clearly distinguish between particles located inside or directly outside membranous structures. Nevertheless, single-cell depth-resolved Raman mapping, reported in Figures S2–S3, showed that the PS-associated spectral signal was concentrated within the same axial region as the maximum cellular CH-stretching signal and decreased outside this region. These observations support localization within the cell-associated volume, although the Raman measurements alone cannot unambiguously distinguish intracellular particles from those closely associated with the basal plasma membrane. Accordingly, throughout this work, we refer to cellular or cell-associated PS accumulation rather than strictly intracellular localization, wherever this distinction cannot be independently established.

To assess the time- and dose-dependent evolution of PS-NPs uptake, multiple Raman maps were acquired for each experimental condition (see Materials and Methods Section). Qualitative examination of NP spatial distributions across maps reveals systematic trends illustrated in Figure 3, which shows the overlaid Raman maps of PS signal on the cellular amide I distribution. After 6 h of incubation (left panels), cells exposed to both 16 and 25 μg/mL PS-NPs exhibit minimal PS-positive puncta randomly distributed, indicating a low initial uptake. By 24 h (right panels), both conditions display increased PS signal relative to 6 h. The 16 μg/mL condition shows a markedly more pronounced accumulation, with numerous PS-positive pixels diffusely distributed across the cellular area. The 25 μg/mL condition also exhibits increased PS signal at 24 h, yet with fewer and more spatially localized puncta compared to the lower dose at the same time point.

**Figure 3:**
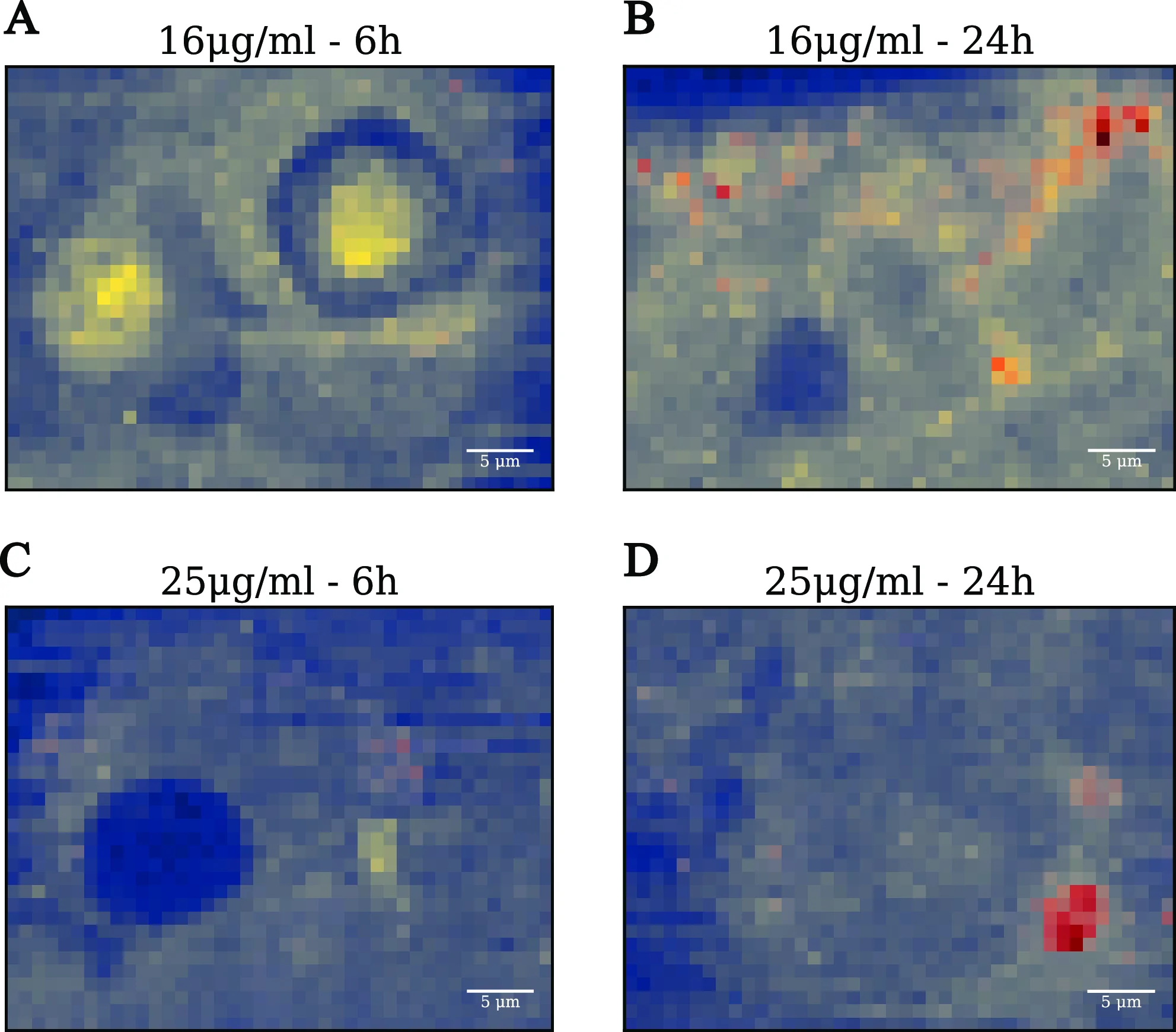
Dose- and time-dependent spatial distribution of PS uptake in Caco-2 cells. Hyperspectral Raman maps showing PS signal (red pixels) overlaid on cellular amide I distribution (yellow-blue scale) for selected experimental conditions, with the same colorbars across the maps. (Top row) Cells exposed to 16 μg/mL PS after (A) 6 h and (B) 24 h of incubation. (Bottom row) Cells exposed to 25 μg/mL PS after (C) 6 h and (D) 24 h of incubation. At early time points (6 h), both concentrations exhibit minimal PS-positive signal, indicating low initial uptake regardless of dose. By 24 h, both conditions display increased PS accumulation: the 16 μg/mL condition shows a markedly pronounced and spatially diffuse distribution of PS-positive pixels across the cellular area, while the 25 μg/mL condition presents fewer and more localized puncta, consistent with concentration-dependent uptake kinetics and a possible earlier saturation of NP uptake at the higher dose. The spatial heterogeneity reflects the subcellular localization of NP aggregates.

The qualitative patterns observed in Figure 3 demonstrate clear dose- and time-dependent variations in PS localization. To actively quantify the PS signal and account for spatial heterogeneity in cellular biochemical composition, we developed a ratiometric approach that balances chemical specificity with robustness to local cellular content variations and intensity fluctuations across different Raman maps. For each preprocessed pixel spectrum in a given map, we numerically integrated the characteristic PS marker band in the 980–1015 cm^–1^ window (*A*_PS,i_) and the cellular amide I band around 1660 cm^–1^ (*A*_amideI,i_). While the former Raman peak corresponds to the phenyl ring “breathing” mode in the PS backbone, and serves as a reliable marker for PS-NPs,^30^ the latter primarily arises from C=O stretching vibrations in the peptide backbone, and is ubiquitous in cellular proteins. The amide I peak is considered as a normalization reference, accounting for the efficiency of optical detection and different turbidity of the buffer solution across the samples. It should be noted that this ratiometric normalization reduces variability but does not provide an absolute measure of particle number or PS mass or fully eliminate matrix-dependent effects (Figure S1), a limitation also acknowledged for fluorescence-based workflows relying on comparable light scattering between matrices.^29^ Such peak’s area was averaged over all cellular pixels within each map, yielding a map-level constant $\overset{®}{A_{amide\;I}^{\mathrm{on}‐\mathrm{cell}}\;}$ that serves as a common denominator for all pixel-wise ratio calculations. This approach ensures that spatial variations in the resulting ratio reflect changes in PS content rather than local protein concentration heterogeneity, and mitigates map-to-map variability associated with experimental acquisition efficiency. The resulting per-pixel ratiometric parameter was then defined as $R_{i}=A_{\mathrm{PS},i}/\overset{®}{A_{amide\;I}^{\mathrm{on}‐\mathrm{cell}}\;}$ , a dimensionless quantity providing a relative measure of PS content normalized to the cellular protein background. Higher *R*_*i*_ values indicate greater PS abundance relative to the cellular matrix.

A critical step in the analysis was establishing an objective criterion to distinguish pixels containing PS-NPs from those representing the cellular background signal. This distinction is nontrivial because cells exhibit an intrinsic Raman signal near 1002 cm^–1^ arising from endogenous phenylalanine content, as shown from the control spectrum in Figure 1. To achieve this, we analyzed Raman maps acquired from unexposed control cells that were processed in parallel with PS-exposed samples. The distribution of pixel-wise ratio *R*_*i*_ was computed using the same procedure described above, and recentered by its mode to remove the baseline offset common to all cells (see Materials and Methods Section); the maximum value of this shifted distribution was taken as the threshold *T*_ctrl_, representing the upper limit of the intrinsic cellular contribution to the 1002 cm^–1^ Raman band. For all subsequent analyses of PS-exposed samples, any pixel whose shifted *R*_*i*_ value exceeded *T*_ctrl_ was classified as PS-positive, indicating the presence of PS aggregates at that location. Conversely, pixels with shifted ratio values below the threshold were classified as PS-negative and attributed to the cellular background. Figure 4 illustrates the classification procedure: panel (A) shows the distribution of *R*_*i*_ values across three experimental conditions (control, 16 μg/mL and 25 μg/mL), demonstrating that PS-exposed samples exhibit a population of pixels with *R*_*i*_ values exceeding the control-derived threshold, while the right panels show a representative spatial map of *R*_*i*_ values, before (B) and after (C) threshold-based classification, for a 25 μg/mL–15 h condition.

**Figure 4:**
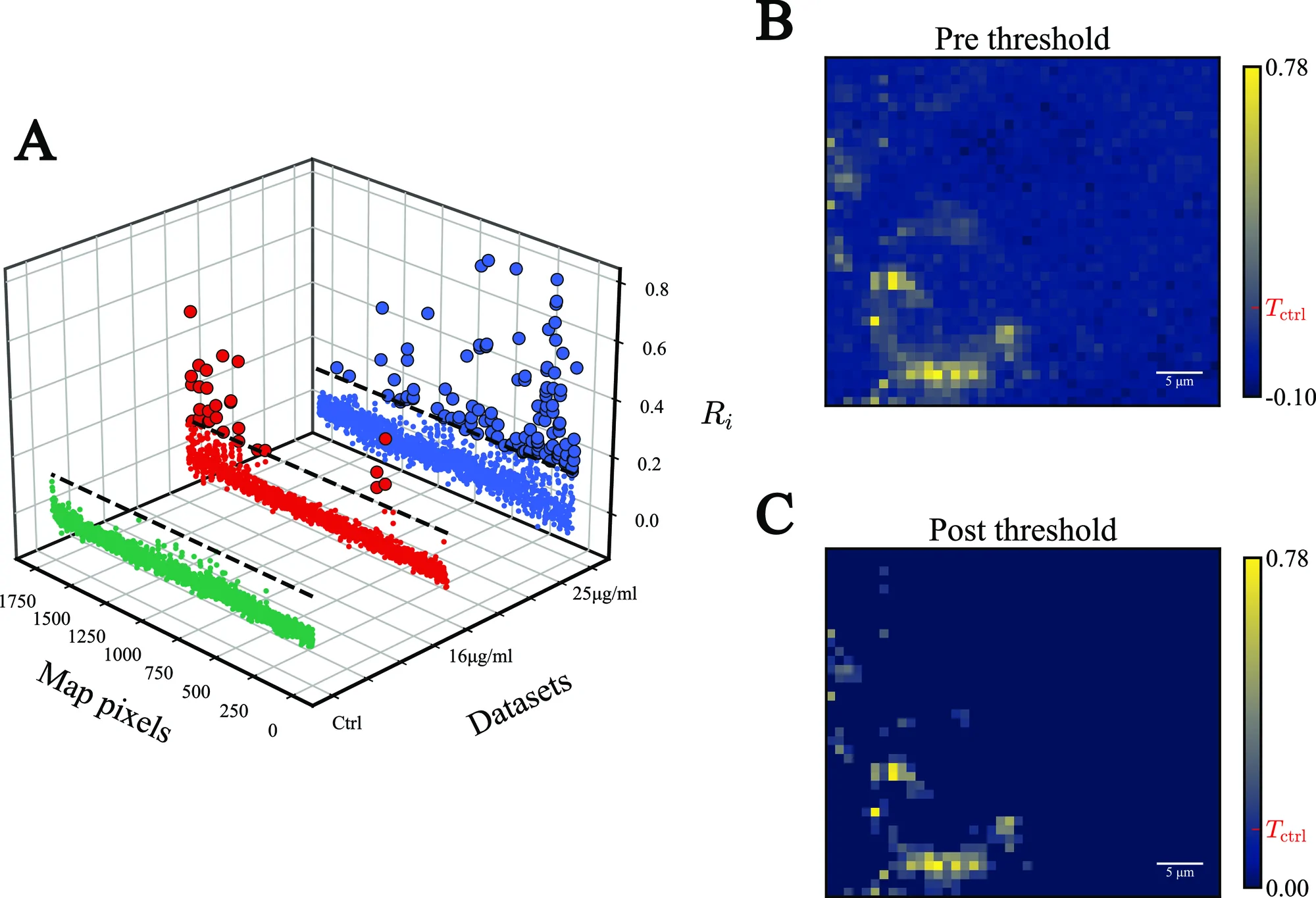
Ratiometric pixel classification and threshold-based PS identification. (A) Three-dimensional plot showing the distribution of pixel-wise ratiometric values $R_{i}=A_{\mathrm{PS},i}/\overset{®}{A_{amide\;I}^{\mathrm{on}‐\mathrm{cell}}\;}$ for three representative data sets of unexposed control cells (green), cells exposed to 16 μg/mL PS-NPs (red), and cells exposed to 25 μg/mL PS-NPs (blue) at 15 h. Control pixels cluster at low *R*_*i*_ values reflecting endogenous phenylalanine contribution, with the maximum defining the discrimination threshold *T*_ctrl_ (dashed line). PS-exposed samples exhibit populations of pixels exceeding the threshold. Representative spatial map of *R*_*i*_ values before (B) and after (C) threshold-based classification. Pixels below the threshold were set to zero, while pixels above the threshold retained their original continuous values. This transformation enables unambiguous identification of NP accumulation sites within the cellular area, and facilitates subsequent per-cell quantification.

In order to quantify the single-cell PS uptake, we developed a semiautomated segmentation procedure to delineate individual cell boundaries within each Raman map. Cells were segmented based on their morphological features visible in the Raman protein and lipid distribution maps (C–H stretching region, 2850–2950 cm^–1^), which provided sufficient contrast to distinguish cell boundaries even in the absence of traditional staining (details in Materials and Methods Section). The resulting filtered map *M*_cell_ contained nonzero entries only at pixel locations corresponding to the interior of that specific cell. Two complementary per-cell descriptors of PS-NPs uptake were then defined. The first, the PS filling fraction (FF_cell_), quantifies the fraction of cellular area occupied by PS-positive pixels:

1 $$FF_{cell}=\frac{1}{N_{cell}}\sum_{\left(j,k\right)\in map}P_{thresh}\left(j,k\right)\cdot M_{cell}\left(j,k\right)$$

where ***P***_thresh_(*j*, *k*) is the threshold matrix, with entries equal to 1 at pixels where *R*_*i*_ > *T*_ctrl_ and 0 elsewhere, *N*_cell_ = ∑_(*j,k*)_ *M*_cell_(*j*, *k*) is the total number of pixels within the cell, and the sum runs over all pixel coordinates (*j*, *k*). The filling fraction is conceptually analogous to area-occupancy metrics used in fluorescence image analysis of intracellular NP accumulation,^15^ and provides a straightforward spatial metric of NP presence that is independent of local signal intensity. The second descriptor, the cell-normalized uptake metric *U*_cell_, integrates both the spatial extent and the local PS signal intensity by summing the thresholded ratiometric values *R*_i_^*^ = *R*_*i*_·*P*_thresh_ over the cellular mask

2 $$U_{cell}=\frac{1}{N_{cell}}\sum_{\left(j,k\right)\in map}R_{i}^{*}\left(j,k\right)\cdot M_{cell}\left(j,k\right)$$

The normalization by cell area enables direct comparison across cells of different sizes, thus cells with more extensive or more intense PS accumulation yield higher *U*_cell_ values. Because *U*_cell_ is normalized both to the mean on-cell amide I signal and to the cell area, it should be understood as a relative index of the PS burden per cell, rather than an absolute measure of particle number or mass; for brevity, we refer to this quantity as the cellular PS burden or load throughout the manuscript, with such relative meaning implied. While FF_cell_ captures the spatial footprint of NP distribution, *U*_cell_ additionally encodes local relative concentration information, making the two descriptors complementary probes of cellular NP accumulation.

The per-cell uptake parameters were computed for each cell in the data set, yielding a distribution of uptake values representative of the population heterogeneity. From these, population means ( ${\mathrm{U̅}}_{cell}$ , ${\overset{®}{\mathrm{FF}}}_{\mathrm{cell}}$ ) and their standard deviations were computed, characterizing the central tendency and variability of PS uptake at that condition, and then plotted as a function of incubation time, with error bars representing the standard error of the mean (see Figure 5A–B). This approach allowed us to quantitatively assess dose- and time-dependent trends in NP accumulation while accounting for cell-to-cell variability inherent in biological systems.

**Figure 5:**
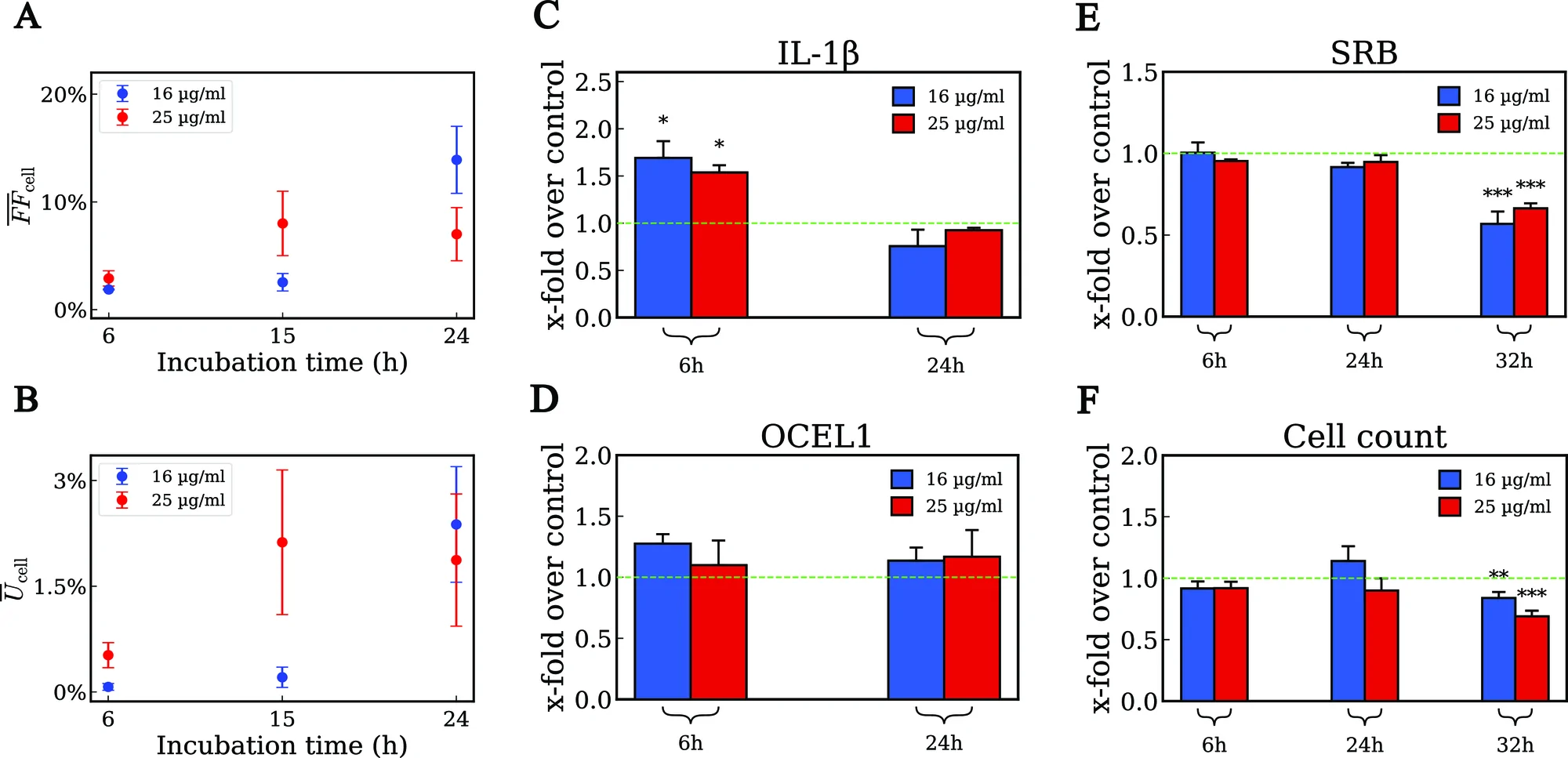
Integrated Raman spectroscopic and biological characterization of PS-NPs uptake dynamics and cellular responses. (A) PS filling factor, quantifying the percentage of cellular area occupied by PS-positive pixels, plotted as a function of incubation time for Caco-2 cells exposed to 16 μg/mL (blue) and 25 μg/mL (red) PS-NPs. (B) Cell-normalized PS uptake metric *U*_cell_, defined as the sum of ratiometric values *R*_*i*_ for all PS-positive pixels normalized by total cell area, plotted as a function of incubation time for Caco-2 cells exposed to 16 μg/mL (blue) and 25 μg/mL (red) PS-NPs. Error bars represent standard errors of the mean across ∼15 individual cells per condition. Both panels reveal concentration-dependent uptake dynamics: the high dose (25 μg/mL) displays an uptake profile peaking at 15 h and plateauing by 24 h, whereas the low dose (16 μg/mL) shows progressive monotonic accumulation throughout the 24 h observation window, reaching its highest recorded value at the final time point. (C) IL-1β expression levels normalized to control, showing early pro-inflammatory response at 6 h that returns to baseline by 24 h for both concentrations (* indicates*p* < 0.05 *vs* control). (D) OCEL1 expression levels, indicating no significant changes in tight junction regulation during the observation window. (E) Cell viability assessed by SRB assay, demonstrating concentration-dependent reduction at 32h (*** indicates *p* < 0.001 *vs* control). **(**F) Direct cell count measurements confirming viability trends, with significant reduction at 32h for both doses (** and *** indicate *p* < 0.01 and *p* < 0.001 *vs* control, respectively). The correspondence between spatial uptake patterns (A–B) and biological end points (C–F) may be in line with an influence of cellular NP distribution on inflammatory signaling and cytotoxic outcomes.

The quantitative PS uptake kinetics is presented in Figure 5. Panel (A) shows the PS filling fraction FF_cell_, quantifying the percentage of cellular area occupied by PS-positive pixels as a function of incubation time. At 6h, both concentrations show minimal PS signal, consistent with the qualitative observations in Figure 3. The two conditions then diverge markedly: cells exposed to 25 μg/mL reach a maximum at 15 h and plateau by 24 h, compatible with accumulation approaching saturation within the experimental time frame, while cells exposed to 16 μg/mLdisplay a progressive, monotonic accumulation throughout the entire observation window, with no detectable decline up to 24 h. This concentration-dependent contrast may suggest that higher extracellular NP concentrations accelerate endocytic accumulation toward an earlier saturation plateau, consistent with a shared underlying mechanism of endocytic accumulation operating at concentration-dependent rates. The earlier saturation plateau at 25 μg/mL likely reflects faster saturation of endocytic pathways or enhanced NP-receptor encounter frequency at higher extracellular concentrations, whereas the monotonic accumulation at 16 μg/mL hints that internalization dynamics dominate over the entire 24 h window, with any putative saturation or subsequent clearance likely extending beyond the experimental time frame. Investigating the underlying uptake pathway was beyond the scope of this study, so the interpretation above remains a plausible hypothesis open to further validation.

In parallel with the label-free Raman-based quantification of NP uptake, we performed complementary *in vitro* assays to investigate early inflammatory and cytotoxic responses to PS-NPs exposure (Figure 5C–F). A modest but statistically significant increase in IL-1β transcript levels was observed at 6h for both concentrations, with levels returning to baseline by 24 h. Given that the magnitude of this transcriptional change remained below the 2-fold threshold, it was interpreted as a transient transcriptional response rather than evidence of a fully established IL-1β-mediated inflammatory response. Furthermore, IL-1β production depends not only on transcriptional induction but also on inflammasome-mediated processing of pro-IL-1β into its mature form. As this mature form showed no significant change, PS-NP exposure under the acute conditions tested may have generated only a weak priming signal, insufficient to trigger the downstream maturation machinery (Figure 5C). This modest response is consistent with NP-induced activation of redox-sensitive pathways, including ROS production and subsequent NF-kB signaling, which are well-established drivers of IL-1β transcription in epithelial systems.^34^ OCEL1 transcript levels remained unchanged following PS-NP exposure (Figure 5D). OCEL1 is an occludin/ELL-domain-containing protein but should not be regarded as a canonical tight-junction marker.^35^ It was selected as an exploratory barrier-associated end point because it has been implicated in cytokine-induced occludin mobilization and internalization, processes that may contribute to epithelial barrier loss.^35^ OCEL1 has also been proposed to promote epithelial apoptosis through increased caspase-3 transcription.^36^ Accordingly, its expression was evaluated to assess whether PS-NP accumulation was associated with early molecular changes potentially linked to epithelial junction remodeling or barrier impairment. Because OCEL1 is not a canonical tight-junction marker, its interpretation was considered alongside more direct structural and functional readouts, including transepithelial electrical resistance (TEER) measurements, which showed no significant differences between PS-NP-treated and control monolayers. Taken together, these findings indicate that, under the acute exposure conditions examined, Raman-detected cellular accumulation was not accompanied by measurable functional impairment of the epithelial barrier. Cell viability analysis, performed by SRB assay and direct cell counting, revealed no significant effects at early time points (6–24 h), but showed a marked reduction at 32h (Figure 5E–F), suggesting a model in which inflammatory activation precedes detectable cytotoxicity.

At first glance, however, the filling fraction data present an apparently counterintuitive result: at 24 h, the higher-dose condition exhibits a lower FF_cell_ than the lower-dose condition (Figure 5A). This observation seemingly conflicts with the expectation that a higher extracellular NP concentration should drive proportionally greater cellular accumulation and is difficult to reconcile, on its own, with the complementary biological assay results.

A second, independent inconsistency emerges when the FF_cell_ profiles are compared with the cytotoxicity end points. Inspection of Figure 5A reveals that at 15 h and 24 h, the 25 μg/mL condition yields FF_cell_ values below 10%, while the 16 μg/mLcondition surpasses this level only at 24 h. Were FF_cell_ to be treated as a direct predictor of cytotoxic threshold, one would expect cell death to manifest selectively (or at least earlier) in the 16 μg/mL group by 24 h. Yet panels ([Fig fig5]E–F) show that cell viability is statistically unaffected at 24 h for both concentrations, with significant cytotoxicity emerging at 32 h regardless of the dose. The FF_cell_ metric alone is therefore insufficient to account for the observed pattern of cellular toxicity.

The cell-normalized uptake metric *U*_cell_ (Figure 5B), which encodes both spatial extent and local PS load, offers a possible explanation for these apparent paradoxes. Panel (B) shows that at 24 h, *U* _cell_ values for the two concentrations are comparable within experimental uncertainty, indicating that the cell-associated PS burden is comparable at this time point. The lower FF_cell_ observed at 25 μg/mL therefore does not reflect a reduced PS burden, but rather a more spatially concentrated cellular distribution: a comparable PS load is confined to a smaller fraction of the cellular area, forming denser and more localized accumulations. Conversely, at 16 μg/mL, a comparable PS burden is distributed more diffusely across the cell, which may enhance its interaction with cellular membranes. This distinction, which is invisible to FF_cell_ alone and hardly accessible to conventional fluorescence-based approaches, is precisely what *U*_cell_ and the combined Raman-based analysis uncover.

The combined Raman-based analysis thus points to two complementary factors that may contribute to NP-induced cellular stress. The first is the cellular PS burden (*U*_cell_), which may relate to the cumulative chemical stress exerted on cells and could contribute to downstream cytotoxic outcomes. The second is the spatial distribution of cell-associated NPs (FF_cell_), which may modulate the degree of subcellular compartmentalization and the local intensity of NP-organelle interactions: a working hypothesis is that at the higher dose (25 μg/mL), accelerated accumulation concentrates NPs into denser, spatially restricted cell-associated deposits, whereas the lower dose (16 μg/mL) generates a more spatially diffuse distribution at 24 h, enabling broader engagement of subcellular compartments and potentially eliciting distinct cellular stress responses.

Despite these distinct spatial accumulation patterns, both doses ultimately converge on comparable cytotoxic outcomes at 32h. This convergence becomes clearer when *U*_cell_ is considered alongside FF_cell_: the former, accessible via label-free confocal Raman microscopy, indicates that the cell-associated NP load is comparable across concentrations at 24 h, reconciling the apparently paradoxical trends in spatial footprint. Neither descriptor alone is, therefore, sufficient to account for the biological responses observed here. This dual-descriptor framework represents a significant methodological advance over conventional fluorescence-based uptake assays, which rely on a single spectrally integrated metric and are inherently prone to signal artifacts such as probe dissociation and photobleaching, precluding the rigorous quantitative comparisons reported here. More broadly, confocal Raman microscopy inherently trades part of the sensitivity, acquisition speed, and field-of-view coverage of fluorescence-based imaging for direct chemical specificity via vibrational fingerprinting, enabling polymer identification without reliance on external labels or fluorescence lifetime differences that can be influenced by the local (cellular) environment.

The concentration-dependent uptake profiles suggest that the two PS doses engage a shared endocytic accumulation machinery operating at markedly different rates: the higher concentration (25 μg/mL) appears to drive accelerated NP accumulation approaching saturation within the 24 h observation window, whereas the lower dose (16 μg/mL) produces a monotonically increasing profile with no detectable plateau within the same time frame. This behavior may arise from several nonmutually exclusive mechanisms: (1) increased encounter frequency between NPs and cellular uptake sites at higher concentrations, leading to faster saturation of endocytic pathways; or (2) dose-dependent saturation of cellular uptake capacity driven by NP burden. The experimental profiles for the higher dose in Figure 5A–B, with peak uptake at 15 h and a plateau by 24 h, are consistent with Caco-2 cells approaching saturation of NP accumulation once a critical cellular load is reached, while the monotonically increasing profile at 16 μg/mLsuggests slower internalization dynamics in which saturation and any subsequent clearance response may emerge beyond the experimental time frame. The plateau at the higher dose may additionally reflect the onset of active exocytosis of internalized NPs, a process reported in other epithelial cell models where such pathways restore cellular homeostasis following transient exposure, with processing efficiency depending on particle load.^37^ These proposed mechanisms offer a promising basis for future mechanistic studies. It is important to note that the cellular effects observed for dispersed PS-NPs should not be directly compared with routine cell growth on bulk polystyrene culture vessels. Cell-culture flasks, dishes, and multiwell plates present continuous macroscopic polystyrene surfaces and do not expose cells to freely dispersed, internalizable particles under standard culture conditions. The observed cellular effects should therefore not be interpreted as evidence that polystyrene is intrinsically cytotoxic in all physical forms, but rather as a consequence of its nanoscale, dispersed, and internalizable presentation.

Taken together, these findings support the idea that both spatial organization and cell-associated PS-NP burden may act as critical and complementary determinants of NP bioactivity, each potentially contributing to the early pro-inflammatory response and delayed cytotoxic outcomes observed across concentrations, with possible implications for the risk assessment of oral nanoplastic exposure scenarios. A further advantage of this label-free, chemically specific detection is its potential applicability to more environmentally realistic coexposure scenarios, in which cells or tissues encounter multiple, chemically distinct nanoplastic populations simultaneously, rather than a single polymer type as modeled in this study.^38^ Differentiation of nanoplastics with distinct polymer backbones (*e.g*., PS *vs* PMMA *vs* PET) should be feasible using their characteristic fingerprint regions, potentially helped by multivariate analysis such as PCA to unmix overlapping spectral contributions. A more challenging case is the coexposure of chemically identical nanoplastics bearing different surface functionalizations (*e.g*., differently charged PS particles),^39^ since such modifications are typically confined to the particle surface and may not produce a sufficiently strong or spectrally isolated Raman signature to allow discrimination based on the core polymer spectrum alone. Addressing this scenario would likely require complementing Raman imaging with other techniques that are sensitive to surface charge or functional groups. Extending the present workflow to these mixed-exposure conditions, spanning both distinct polymer types and distinct surface chemistries, represents a promising direction for future work.

## Conclusions

We have demonstrated a robust, label-free methodology for single-cell quantification of polystyrene nanoparticle uptake in *in vitro* epithelial cells using confocal micro-Raman spectroscopy. By combining spectral preprocessing, ratiometric normalization, control-derived thresholding, and per-cell masking, we addressed the challenge of spectral overlap between PS markers and endogenous cellular signals, enabling identification and quantification of cell-associated nanoparticles. Application of the workflow to Caco-2 cells exposed to two PS concentrations over 24 h revealed distinct concentration-dependent uptake kinetics, which provide quantitative evidence of dose-dependent accumulation dynamics, with the higher-dose profile reaching a plateau consistent with possible saturation within the experimental time frame. Integration with complementary biological assays suggests that both spatial organization and the cell-associated PS load of internalized nanoparticles may be linked to early cellular transcriptional responses and cytotoxic outcomes with potential implications for understanding nanoplastic bioaccumulation and toxicity mechanisms. The Caco-2 model employed in the present study serves as an established *in vitro* proxy for the intestinal epithelial cells encountered by nanoparticles following oral exposure, providing benchmark data directly relevant to risk assessment for oral nanoplastic exposure scenarios through quantitative uptake kinetics and spatial-distribution analysis. However, it should be noted that undifferentiated Caco-2 cells represent a simplified epithelial system and do not fully recapitulate the structural and functional complexity of the *in vivo* intestinal barrier, including mucus production, immune interactions, and tight junction maturation; therefore, further studies using differentiated monolayers or more advanced coculture models will be required to extend these findings to physiologically relevant conditions.

The methodology reported here represents a Raman-based assessment of PS-NP cell-associated uptake kinetics at the single-cell level, bridging the gap between qualitative localization and quantitative uptake analysis. By providing a robust, chemically specific, and label-free workflow for cellular particle quantification with spatially resolved distribution mapping, this work advances the application of Raman microscopy in micro- and nanoplastic research. While this framework offers a foundation for studying nanoparticle–cell interactions, its application to other polymer systems or *in vivo* models represents a promising future direction. The general workflow proposed is not inherently specific to polystyrene and may in principle be transferable to other polymer nanoparticles or more complex biologically relevant exposure scenarios, provided that a suitable Raman marker band and corresponding detection limit are identified for each new polymer or matrix.

From a nanotoxicological perspective, the observation that both dose levels trigger early pro-inflammatory responses, with concentration-dependent differences in cell-associated NP accumulation dynamics, suggests that intestinal epithelial cells may possess active mechanisms to mitigate nanoplastic exposure. However, the correspondence between spatial distribution patterns and biological outcomes highlights that risk assessment must consider not only PS uptake burden but also cellular localization and compartmentalization – mechanistic information that is hardly accessible to conventional fluorescence microscopy, where probe photophysical instability and signal diffusion preclude the quantitative spatial resolution demonstrated here.

## Materials and Methods

### Chemicals

Polystyrene nanoparticles (150 nm, CAS PS-R-0.15, batch PS-R-KM08–2 microParticles) were obtained from microParticles GmbH (Berlin, Germany). Phosphate-buffered saline (PBS) was purchased from Corning (Mediatech, Manassas, VA, USA). Poly-l-lysine (CAS 25988–63–0), sulforhodamine B (SRB) sodium salt (CAS S1402–1G), formalin (37%), trypan blue, trichloroacetic acid (≥99% purity), and Tris base (≥99.9% purity) were purchased from Sigma-Aldrich (Milan, Italy). Sterile circular glass coverslips (9.161 064) were obtained from Exacta Labcenter (Modena, Italy).

### Cell Culture and NPs Exposure

Caco-2 cells (ATCC, Manassas, VA, USA) were maintained in high-glucose Dulbecco’s modified Eagle medium (DMEM; Sigma-Aldrich) supplemented with 10% fetal bovine serum (FBS; Sigma-Aldrich), 1% l-glutamine, 1% penicillin-streptomycin, and 1% nonessential amino acids (all from Sigma-Aldrich), at 37 °C in a humidified atmosphere containing 5% CO_2_. Cells were cultured in 25 and 75 cm^2^ tissue culture-treated Falcon flasks (Corning, Glendale, AZ, USA) and used at passage 14; cell growth was monitored daily, and cells were subcultured at 80–90% confluence to prevent monolayer differentiation. All experiments were performed on undifferentiated Caco-2 cells seeded at a density of 2·10^4^ cells/cm^2^. After 48 h of seeding, cells were exposed to PS-NPs at nominal concentrations of 16 and 25 μg/mL. Nonfunctionalized polystyrene nanoparticles (150 nm diameter, SD = 3 nm) were supplied as an aqueous suspension (5% w/v). According to the manufacturer, the particles were spherical and hydrophobic, and contained no fluorescent label or specific surface functionalization. The selected PS-NP concentrations were chosen to fall within the range commonly employed in previous *in vitro* studies of PS-NP uptake and toxicity in epithelial cell models,^40–44^ balancing sufficient Raman signal intensity for reliable single-cell detection against avoidance of overt acute cytotoxicity within the exposure window. For confocal micro-Raman spectroscopy analyses, cells were seeded in 24-well plates (Sarstedt AG & Co. KG, Numbrecht, Germany, ref 83.3922) on sterile coverslips (pure D263 M borosilicate glass, 12 mm diameter, 0.13–0.16 mm thickness) pretreated with poly-l-lysine and treated as described above. Samples were collected at 6, 15, and 24 h post-treatment; untreated control wells were processed in parallel at each time point. At each collection time, cells were briefly washed with PBS to remove residual culture medium and then fixed with 4% paraformaldehyde (PFA); after fixation, cells were washed three times with PBS to remove PFA residues and transferred to the spectroscopy laboratory for Raman analysis. For gene expression analyses, cells were seeded in 6-well plates and harvested after 6 and 24 h of treatment. For cell viability assessment by SRB assay, cells were seeded in 96-well plates (Sarstedt AG & Co. KG, ref 83.3924) at 2·10^4^ cells/cm^2^; for direct cell counting, cells were seeded in 6-well plates (Sarstedt AG & Co. KG, ref 83.3920). In both cases, cells were harvested at 6, 24, and 32 h postexposure.

### Gene Expression Analysis

Total RNA was isolated from Caco-2 cells using TRIzol Reagent (Invitrogen, CA, USA). RNA concentration and purity were determined using a NanoDrop 2000 spectrophotometer (Thermo Fisher Scientific, DE, USA). cDNA synthesis was performed using 5× All-In-One RT MasterMix (Applied Biological Materials Inc., BC, Canada). Real-time PCR was carried out on a CFX Opus 96 Real-Time PCR System (Bio-Rad, CA, USA) using SsoAdvanced Universal Probes Supermix (Bio-Rad) with gene-specific probes for interleukin-1β (*IL-1*β, ID: qHsaCIP0033362) and occludin-like ELL-domain-containing protein 1 (*OCEL1*, ID: qHsaCEP0024714). Gene expression was normalized to β-actin (*ACT*β, ID: qHsaCEP0036280) as the reference gene, and relative expression was calculated by the 2^–*Δ*Δ*C*_*t*_^ method.

### Cell Viability Assay

Cell viability was assessed by the sulforhodamine B (SRB) colorimetric assay following the protocol of Voigt^45^ with minor modifications. Cultured cells were fixed with 10% (w/v) trichloroacetic acid at 4 °C and subsequently stained for 30 min with 0.4% (w/v) SRB dissolved in 1% acetic acid. Unbound dye was removed by repeated washes with 1% acetic acid, and protein-bound dye was solubilized in 10 mM Tris base. Absorbance was measured at 550 nm using an Infinite 2000 PRO microplate reader (Tecan). All values were normalized to the untreated control.

### Cell Counting

For direct cell enumeration, cells were detached using trypsin and resuspended in 500 μL of culture medium. The cell suspension was diluted 1:1 (v/v) with trypan blue dye, and the total number of viable cells was determined using a Thoma hemocytometer. Values were normalized to the untreated control.

### Statistical Analysis

Sample sizes were determined on the basis of previous *in vitro* studies. All statistical analyses were performed using GraphPad Prism. Data are expressed as mean ± standard deviation (SD); *p* < 0.05 was considered statistically significant. For data sets that satisfied the normality criterion and comprised more than five biological replicates, parametric tests were applied; otherwise, nonparametric alternatives were used. SRB assay and cell count data were analyzed by one-way ANOVA followed by Tukey’s post hoc test for multiple comparisons. Gene expression data were analyzed using the nonparametric Kruskal–Wallis test.

### Micro-Raman Experiment

Micro-Raman measurements were carried out using a confocal Raman microscope (HORIBA Jobin Yvon Labram) equipped with a continuous wave (CW) laser operating at 532 nm with an output power of 17 mW at the sample. The excitation beam was focused onto the sample with a water-immersion objective (Olympus LUMPlanFL N 60×, NA = 1.00) mounted on an upright microscope; scattered light was collected in a backscattering geometry through the same objective. A 100 μm confocal pinhole and 200 μm spectrometer slit were used. Diffused light was then dispersed by a 600 gr/mm diffraction grating and detected using a front-illuminated, thermoelectrically cooled CCD camera (Horiba SYNAPSE, 1024 × 256 pixels). The system provides an in-plane spatial resolution below 1 μm and a spectral resolution of 1.34 cm^–1^. Prior to measurements, the spectrometer was calibrated using the first-order Raman peak of a Silicon standard, aligning it at 521 cm^–1^ to ensure accurate spectral positioning. Raman maps were acquired with an integration time of 1 s per spectrum and a lateral pixel step size of 1 μm. Prior to each Raman map acquisition, a rapid z-scan was performed to identify the focal plane corresponding to the maximum cellular Raman intensity (integrated over the CH-stretching region, 2780–3035 cm^–1^), which was set as the *z*-reference baseline and corresponds to the bulk center of the cell body. To ensure that detected PS signals originate from cell-associated particles rather than surface-adsorbed material, the focal plane was further shifted downward by approximately 3 μm relative to this baseline.

### Raman Preprocessing Pipeline

Raw hyperspectral data sets were processed using custom Python scripts developed for this study (representative raw spectra shown in Figure S5). Each spectrum within a map was subjected to a standardized preprocessing pipeline designed to isolate spectral features of interest from instrumental artifacts and background contributions. Steps were applied systematically across all data sets, ensuring consistency across experimental conditions and time points. First, baseline subtraction was performed using the Adaptive Reweighted Penalized Least-Squares algorithm,^46^ to remove the slowly varying autofluorescence and amorphous glass substrate Raman backgrounds, while preserving Raman features from the cellular environment. Subsequently, cosmic ray spikes arising from high-energy particle events hitting the CCD detector were detected and removed via a first-derivative-based outlier routine. Spike regions were replaced by local interpolation to preserve spectral continuity. Finally, residual background contributions, including the underlying glass substrate signal, were subtracted from the characteristic polystyrene peak using a local linear baseline, estimated per spectrum by interpolating the mean intensities of two narrow flanking regions (975–985 cm^–1^, 1015–1025 cm^–1^), ensuring that polystyrene and cellular peaks were clearly resolved and free from substrate artifacts (illustrated for a representative map in Figures S6–S7).

### Ratiometric Thresholding and PS-Positive Pixel Classification

For each preprocessed spectrum, the per-pixel ratiometric parameter *R*_*i*_ was computed as described above. To classify PS-positive pixels while accounting for the systematic baseline contribution arising from endogenous cellular phenylalanine (present in both control and PS-exposed cells regardless of PS content), the *R*_*i*_ histogram of each map was first shifted by its mode, representing the typical PS-free baseline level within the cell population. This recentering procedure causes PS-free spectra to cluster around zero, removing the baseline offset common to all cells. The classification threshold *T*_ctrl_ was then defined as the maximum value of this shifted distribution observed in an unexposed control sample known to contain no PS (*T*_ctrl_ = 0.138), corresponding to a distance of 8σ from the median of the shifted control distribution (Figure S4). An independent control sample, acquired separately, yielded a comparable threshold (0.135, at 6.2σ from the median) supporting the reproducibility of this cutoff across independent control measurements. Any pixel in a PS-exposed map whose shifted ratio exceeded *T*_ctrl_ was classified as PS-positive; all remaining pixels were classified as PS-negative and attributed to cellular background.

### Single-Cell Segmentation

Cell contours were traced manually using a custom interactive Python algorithm that allowed the user to click on sequential points along the cell perimeter while visualizing these biochemical contrast maps. Upon completion of the contour, a binary mask was created mapping physical coordinates to pixel indices: all pixels located within the traced boundary were assigned a value of 1, whereas all pixels outside the boundary were set to 0. The resulting binary masks delineated the spatial extent of individual cells within the Raman map, with the number of segmented cells per map typically ranging from 3 to 10, depending on the cell sizes and map dimensions. The binary mask corresponding to a given cell was multiplied elementwise with the ratiometric map *R*_*i*_, effectively isolating the ratio values within the cell boundary while setting all external pixels to zero.

## Supplementary Material



## Notes

Raman spectra of PS-NPs in aqueous solution, air, and biological environment; depth-resolved Raman mapping of PS-associated and cellular CH-stretching signals; distribution of shifted ratio values and classification threshold; representative raw Raman spectra of cells, substrate, and pure PS; raw and preprocessed hyperspectral Raman maps and main processing steps (PDF)
