# Supplementary material for: Quantifying Cellular Nanoplastic Accumulation Using Raman Microscopy
Source: ACS Omega. 2026 Oct 22;11(43):66784–94. doi: 10.1021/acsomega.6c06443 (PMC13737012; doi:10.1021/acsomega.6c06443)
Supplement: Supplementary file 1 [file ao6c06443_si_001.pdf]

Supporting information of:

Quantifying cellular nanoplastic accumulation using Raman microscopy

Federico Perrella[1], Valentina Iezzi[2], Roberta Maria Ferri[2], Giulia Fioravanti[1], Marilena Briglia[3], Andrea Marini[1], Elisabetta Benedetti[2], Paola Benassi[1], Carino Ferrante[4*], Davide Tedeschi[1*]

[1]Department of Physical and Chemical Sciences,

University of L'Aquila, Via Vetoio, 67100 L'Aquila, Italy

[2]Life, Health and Environmental Sciences,

University of L'Aquila, Via Vetoio, 67100 L'Aquila, Italy

[3]Department of Medicine and Surgery,

University of Enna "Kore", 94100 Enna, Italy

[4]CNR-SPIN, c/o Department of Physical and Chemical

Sciences - Via Vetoio, 67100, L'Aquila, Italy

[*]carino.ferrante@spin.cnr.it and [*]davide.tedeschi@univaq.it

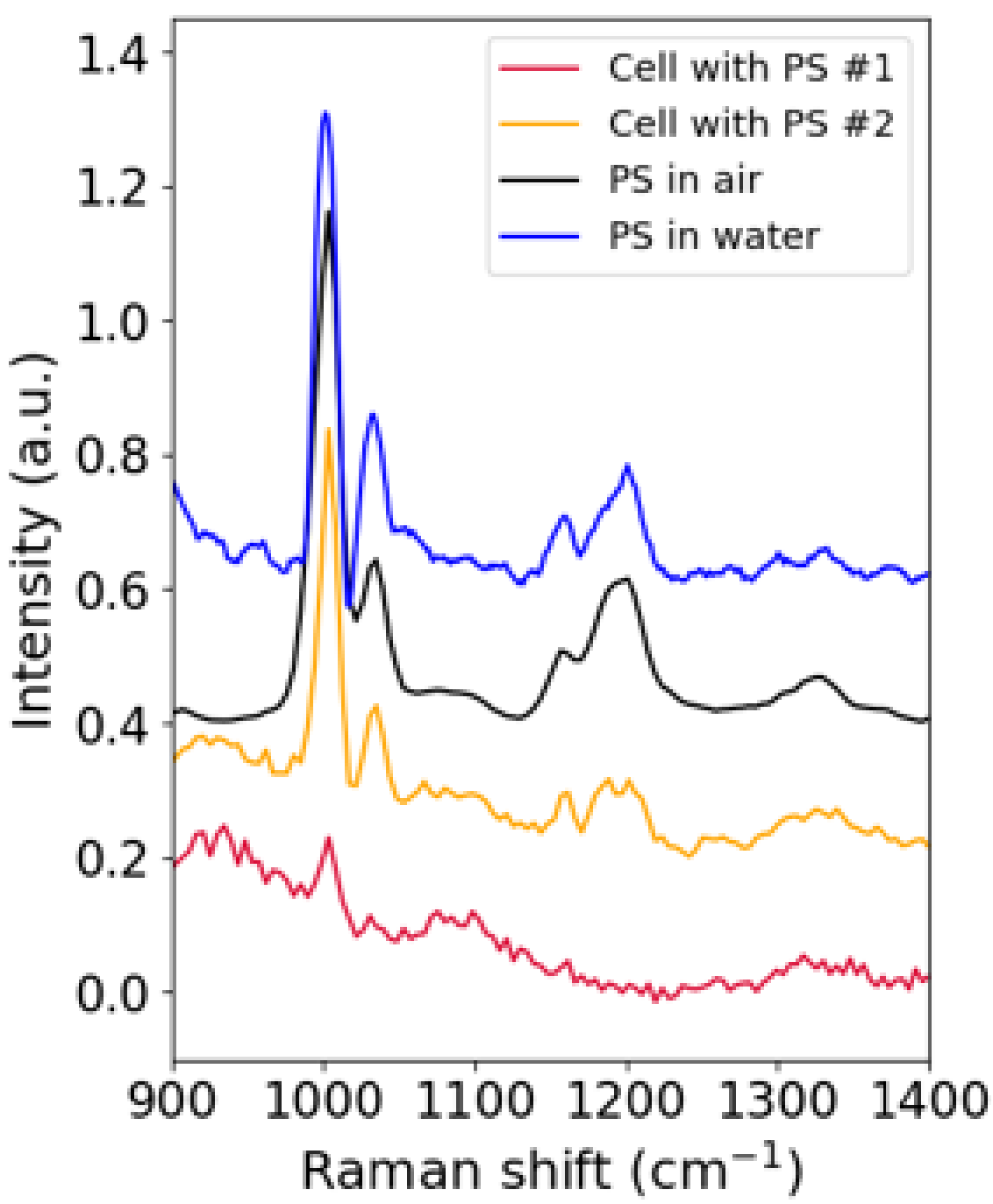

Figure S1   Raman spectra of PS-NPs acquired in aqueous solution, in air, and in a biological environment.

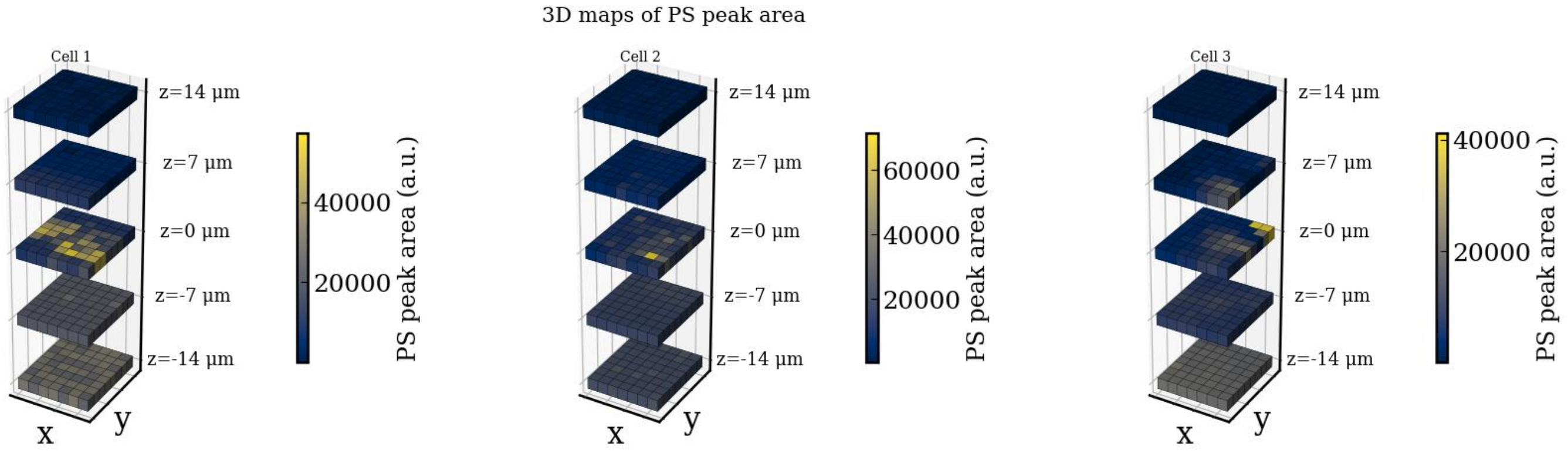

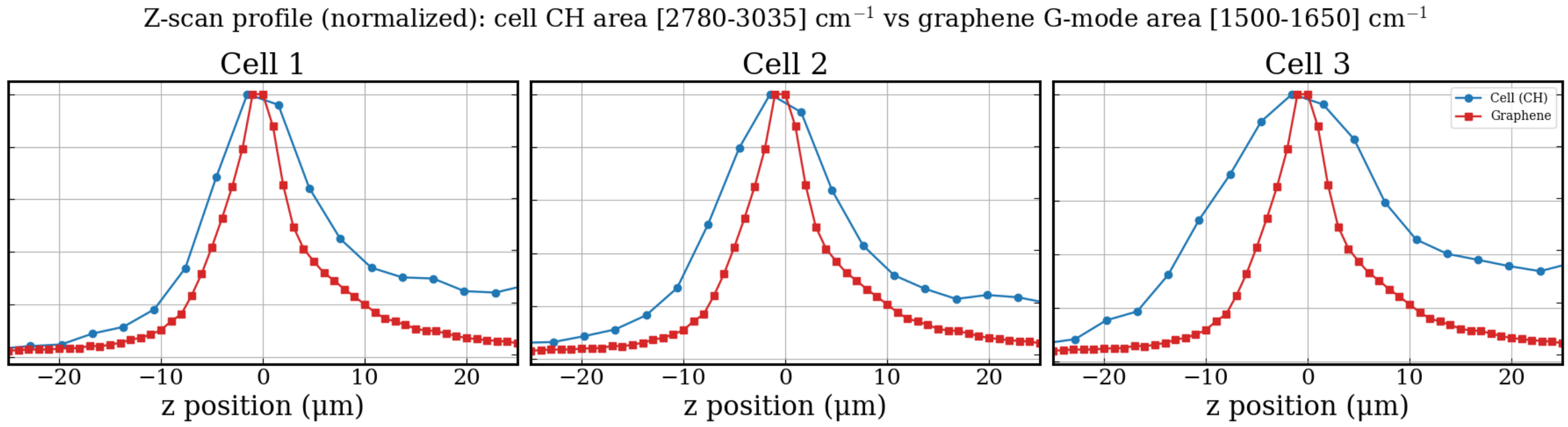

Figure S2 ***Depth-dependent distribution of the PS-associated Raman signal and cellular CH-stretching intensity. Top****: XY Raman maps of the integrated area of the PS ring-breathing band at approximately 1000 cm^{-1}, acquired from three representative cells at five focal-plane positions. For each cell, z=0 corresponds to the focal position associated with the maximum cellular CH-stretching intensity.* ***Bottom****: integrated Raman intensity in the cellular CH-stretching region (blue) and in the graphene G-mode region (red) plotted as a function of the axial displacement from this focal position. Graphene z-scans were acquired to show the instrument axial response function. The characteristic PS-associated signal is strongest within the CH-rich axial region and decreases in focal planes located above and below this region. The apparent signal increase at z = -14 µm reflects the broad glass background contribution near 1000 cm^{-1} rather than an actual PS-related signal, as this focal plane approaches the coverslip surface (as testified by the spectral analysis in Figure S3).*

Representative spectra, zoomed on PS peak (~1000 cm^{-1}) and CH stretch (~2900 cm^{-1})

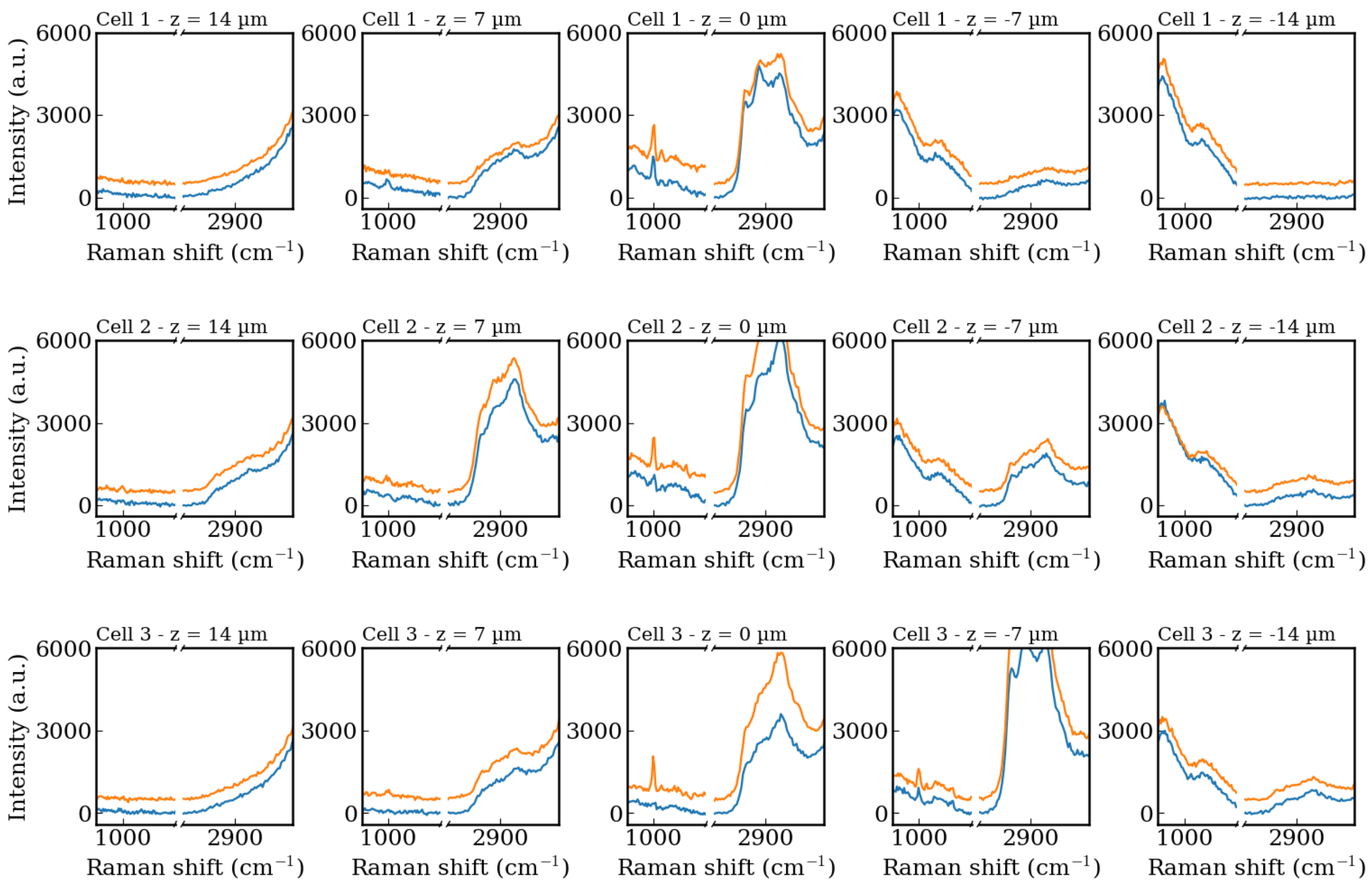

Figure S3 Two different spectra from three representative cells are shown in the regions of the characteristic PS band at approximately 1000 cm^{-1} and the cellular CH-stretching bands at approximately 2900 cm^{-1}. The sharp PS spectral contribution is most evident in the focal planes corresponding to the CH-rich cellular region and decreases or becomes spectrally indistinguishable from the substrate background at focal positions outside this region.

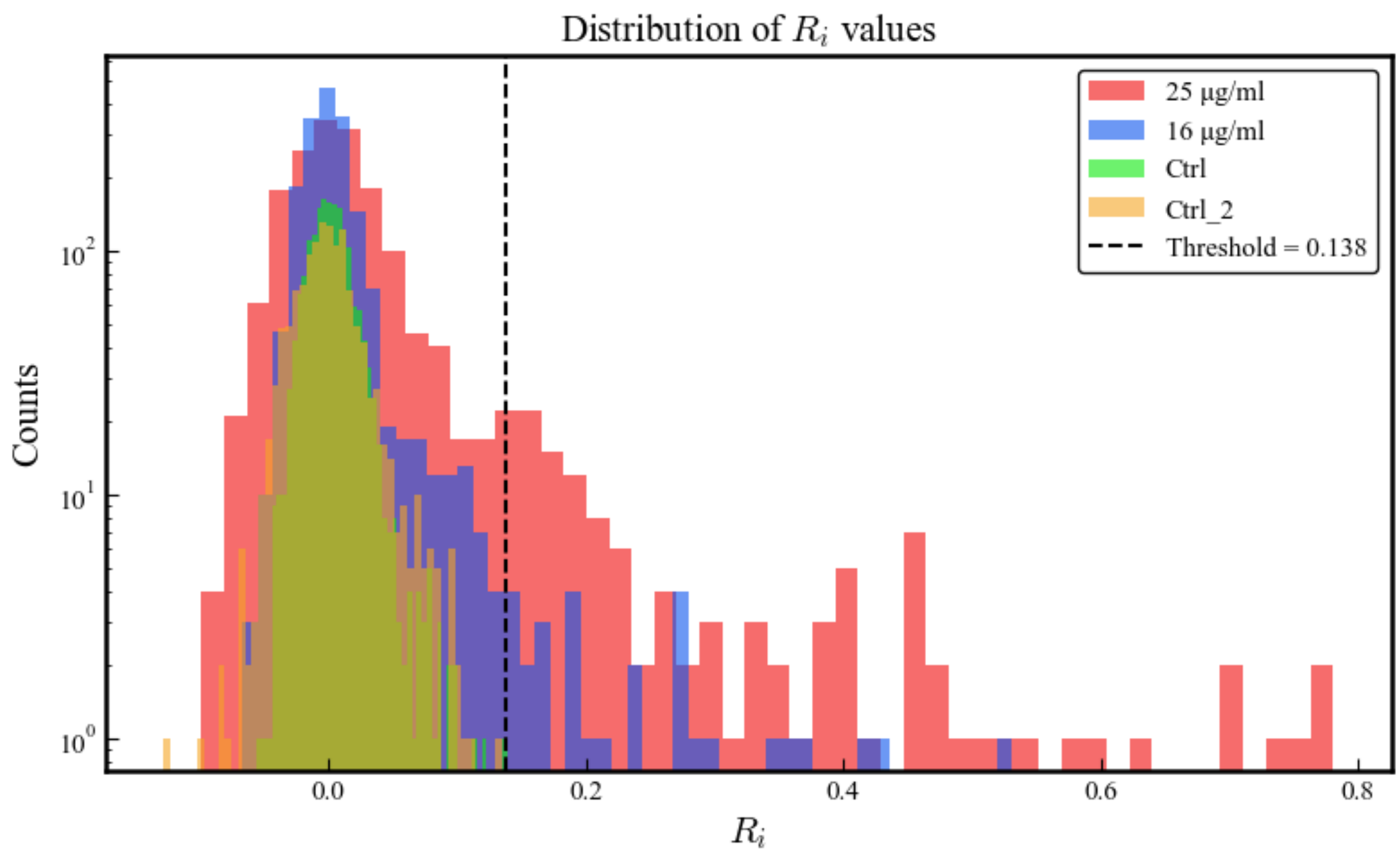

Figure S4 Log distribution of shifted R_i values for control (Ctrl and Ctrl_2, green and yellow) and 15h PS-treated cell populations at 16 µg/ml (blue) and 25 µg/ml (red). The dashed line marks the classification threshold (R_i = 0.138, sitting 8σ away from the median). Spectra exceeding this threshold are classified as PS-positive.

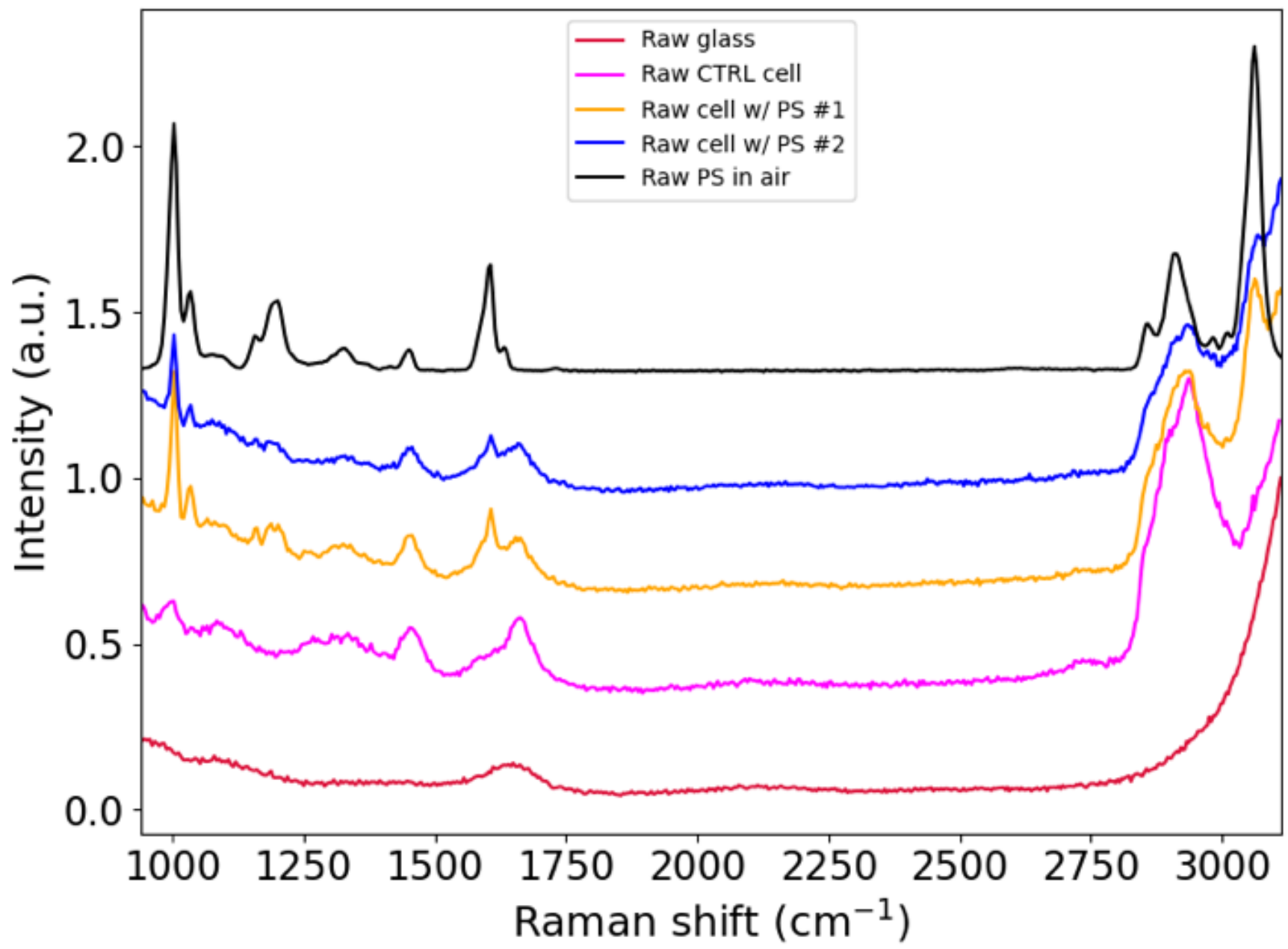

Figure S5 Representative raw (unprocessed) normalized Raman spectra of control cells, PS-contaminated cells, substrate glass and pure PS.

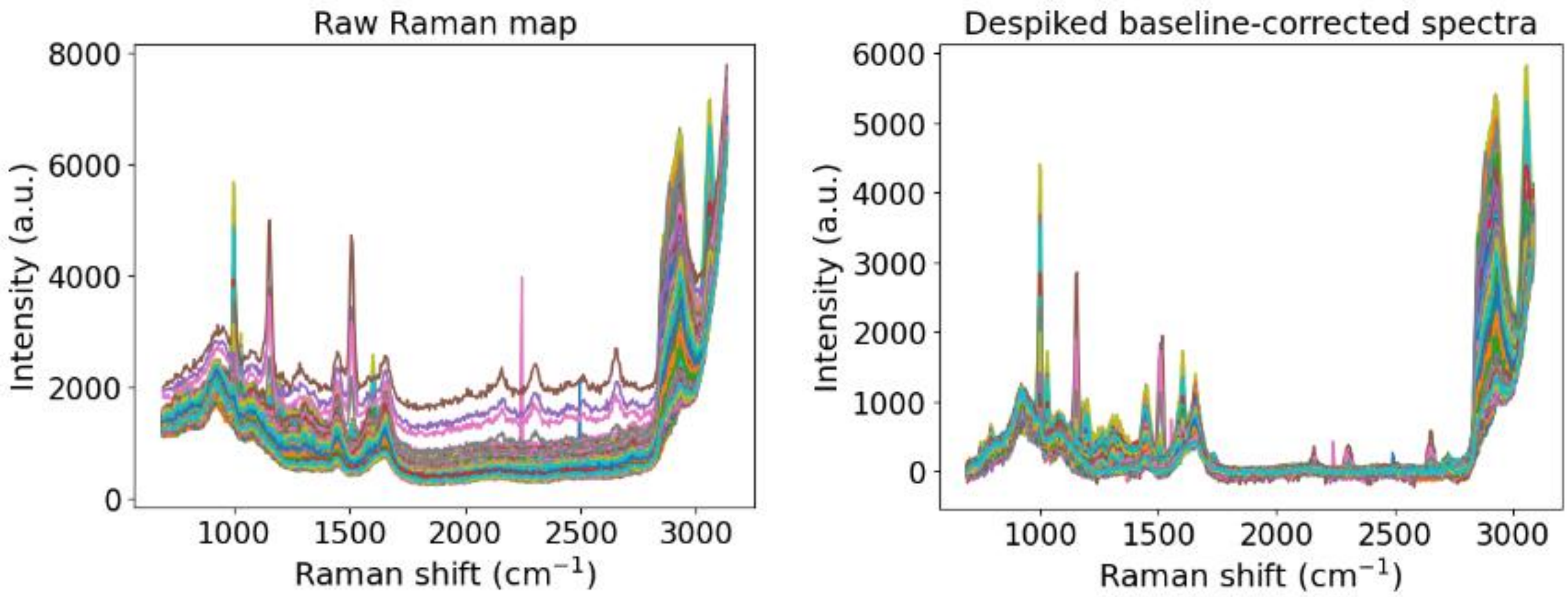

Figure S6 Raw (left) and pre-processed (right) hyperspectral Raman map after baseline-subtraction and despiking procedure.

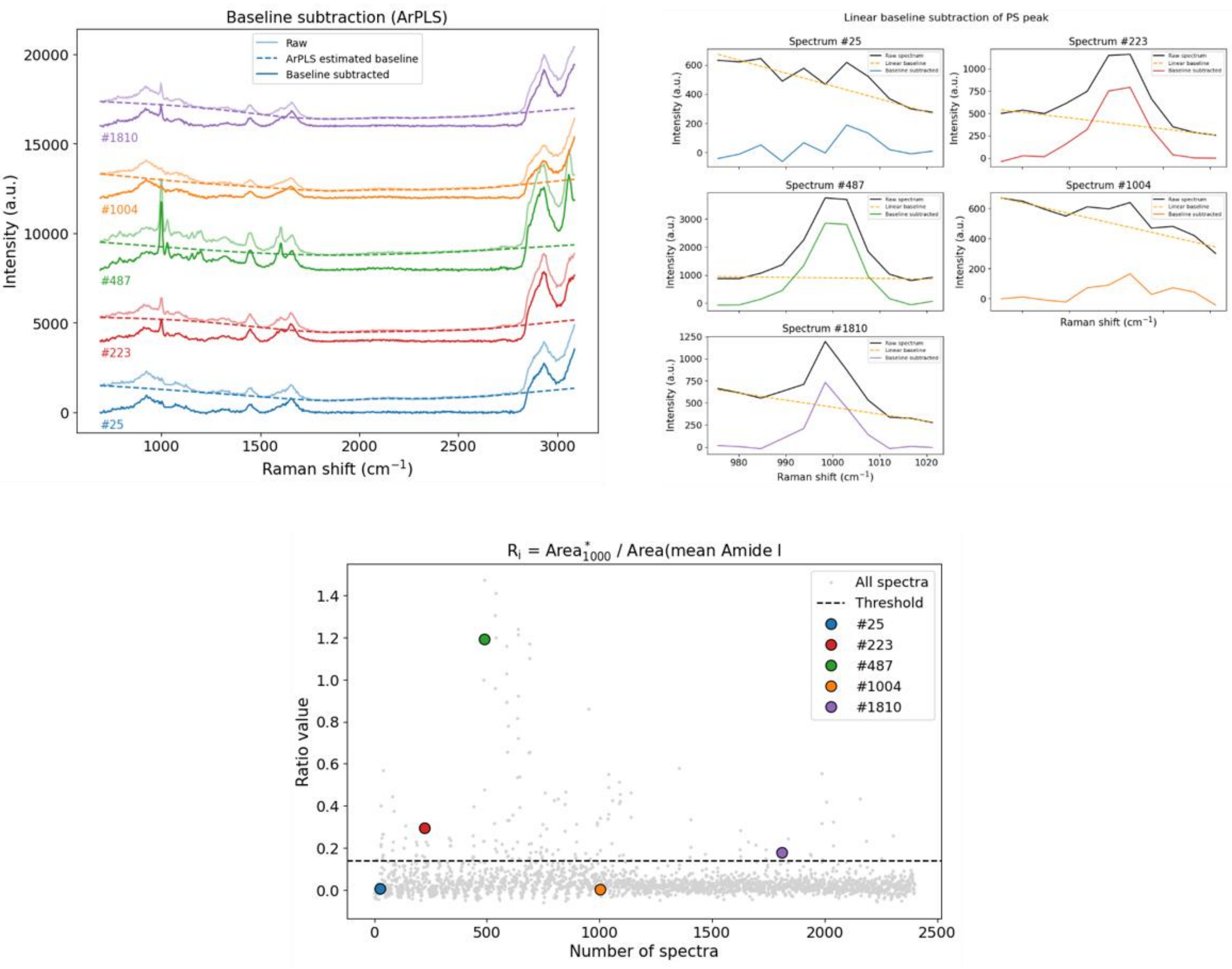

Figure S7 Main processing steps of a hyperspectral Raman map.

(Top left) Examples of raw spectra, ArPLS estimated baselines and corresponding baseline-subtracted spectra.

(Top right) Additional (linear) baseline-subtraction for the PS peak, before classification.

(Bottom) R_i distribution of the considered map, with coloured dots highlighting the selected Raman spectra shown.
